# Distribution of antibodies against influenza virus in pigs from farrow-to-finish farms in Minas Gerais state, Brazil

**DOI:** 10.1111/irv.12304

**Published:** 2015-04-23

**Authors:** Alessandra S Dias, Érica A Costa, Daniela S Rajão, Roberto M C Guedes, Janice R Ciacci Zanella, Zélia I P Lobato

**Affiliations:** a Laboratório de Pesquisa em Virologia Animal, Departamento de Medicina Veterinária Preventiva, Universidade Federal de Minas GeraisBelo Horizonte, MG, Brazil; b Virus and Prion Disease of Livestock Research Unit, USDA-ARSAmes, IA, USA; c Departamento de Clínica e Cirurgia Veterinárias, Escola de Veterinária, UFMGBelo Horizonte, MG, Brazil; d Embrapa Suínos e AvesConcórdia, SC, Brazil

**Keywords:** Antibodies, Brazil, H3N2, pandemic H1N1, serological profile, swine influenza

## Abstract

**Background:**

Swine influenza virus (SIV) is the cause of an acute respiratory disease that affects swine worldwide. In Brazil, SIV has been identified in pigs since 1978. After the emergence of pandemic H1N1 in 2009 (H1N1pdm09), few studies reported the presence of influenza virus in Brazilian herds.

**Objectives:**

The objective of this study was to evaluate the serological profile for influenza virus in farrow-to-finish pig farms in Minas Gerais state, Brazil.

**Methods:**

Thirty farms with no SIV vaccination history were selected from the four larger pig production areas in Minas Gerais state (Zona da Mata, Triângulo Mineiro/Alto Paranaíba, South/Southwest and the Belo Horizonte metropolitan area). At each farm, blood samples were randomly collected from 20 animals in each production cycle category: breeding animals (sows and gilts), farrowing crate (2–3 weeks), nursery (4–7 weeks), grower pigs (8–14 weeks), and finishing pigs (15–16 weeks), with 100 samples per farm and a total of 3000 animals in this study. The samples were tested for hemagglutination inhibition activity against H1N1 pandemic strain (A/swine/Brazil/11/2009) and H3N2 SIV (A/swine/Iowa/8548-2/98) reference strain.

**Results:**

The percentages of seropositive animals for H1N1pdm09 and H3N2 were 26·23% and 1·57%, respectively, and the percentages of seropositive herds for both viruses were 96·6% and 13·2%, respectively.

**Conclusions:**

The serological profiles differed for both viruses and among the studied areas, suggesting a high variety of virus circulation around the state, as well as the presence of seronegative animals susceptible to influenza infection and, consequently, new respiratory disease outbreaks.

## Introduction

Swine influenza virus (SIV) is the cause of an acute respiratory disease that affects swine worldwide. SIV infection is characterized by fever, inactivity, decreased food intake, respiratory distress, coughing, sneezing, conjunctivitis, and nasal discharge.^1^,^2^ Disease severity is affected by many factors, including the viral strain, but typically, the onset of disease is sudden. The incubation period is between 1 and 3 days, with rapid recovery beginning 4–7 days after onset. The disease is characterized by high morbidity and generally low mortality rates.^3^

Influenza A viruses are members of the *Orthomyxoviridae* family and are 80–120 nm enveloped viruses with segmented, single-stranded, negative-sense RNA genomes.^3^ The segmented genome of influenza virus allows reassortment between different viruses, and once cells are infected with two or more different influenza viruses, the exchange of RNA segments between the viruses allows the generation of progeny containing a novel combination of genes.^3^ The viral surface glycoproteins hemagglutinin (HA) and neuraminidase (NA) are the main targets of the host immune response, and they are important for host specificity and virulence.^4^ HA binds to the cell receptor N-acetylneuraminic acid-2,3-galactose linkage or to the N-acetylneuraminic acid-2,6-galactose linkage on sialyloligosaccharides of avian and mammalian viruses, respectively.^5^ Swine have been considered to be a potential ‘mixing vessel’ because they have receptors for both avian and human influenza viruses.^6^

In 2009, a new influenza virus emerged in the human population of North America. Pandemic H1N1 (H1N1pdm09), which has a unique genome with six gene segments (PB1, PB2, PA, HA, NP, and NS) from the triple reassortant swine lineage of the North American virus and the M and NA gene derived from the Eurasian lineage of the swine influenza virus,^7^ had never before been recognized in swine. Immediately after the spread of H1N1pdm09 in human populations, outbreaks in pigs were reported in many countries worldwide.^8^

Brazil is the fifth leading global pork producer and the fourth largest pork exporter, and swine production is economically important. However, few studies have investigated the presence of SIV antibodies or virus isolates in Brazilian pigs. In Brazil, SIV was first isolated in 1978 in a pig from Minas Gerais state.^9^ One study reported a low prevalence of antibodies against H1N1 and H3N2 subtypes in pigs from 10 Brazilian states between 1996 and 1999.^10^ Further studies demonstrated the prevalence of anti-influenza antibodies against human^11^ and swine viruses^12^ in southeastern Brazil. In a seroprevalence study in Paraná (southern Brazil), the authors reported that 46% of the sampled farms were positive for anti-H3N2 antibodies, and the prevalence of antibodies against human H3N2 in those pigs was 20%.^13^

After the H1N1pdm09 outbreak, few studies reported the presence of influenza virus in Brazilian herds.^14^,^15^ However, no data are available concerning the prevalence of antibodies against swine influenza virus in Brazilian herds after 2009. In Brazil, a vaccine protecting swine against influenza virus was licensed on May 2014. Prior to that vaccine, the presence of anti-influenza antibodies in pigs was attributed to natural infection. Recently, there were many reports regarding respiratory outbreaks in farms around the country^16^ and producers and veterinarians began vaccinating against swine influenza to reduce economic losses.

An analysis of the serological profile may provide information regarding viral circulation and might be useful in implementing vaccination strategies and effective control measures based on the characteristics of individual herds. Thus, the objective of this study was to evaluate the serological profile for influenza virus in pigs from farrow-to-finish farms in Minas Gerais state, Brazil.

## Materials and methods

### Sample collection

**Figure 1:**
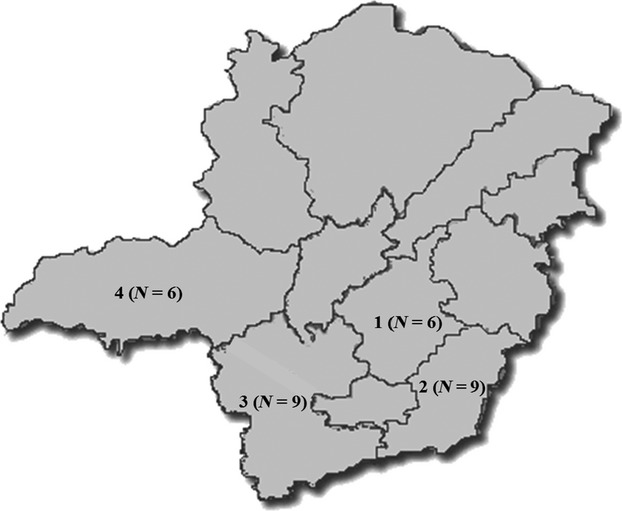
Minas Gerais state map with all sampled areas in this study: 1 = Belo Horizonte Metropolitan area; 2 = Zona da Mata; 3 = South/Southwest; and 4 = Triângulo Mineiro/Alto Paranaíba. *N* = number of sampled farms in each area.

Serum samples, which were collected from May to August 2012 by jugular puncture, were centrifuged after clot formation, and the serum was stored at −20°C. Thirty farms (F1 to F30) from the four larger pig production areas in Minas Gerais state (Zona da Mata, Triângulo Mineiro/Alto Paranaíba, South/Southwest and the Belo Horizonte metropolitan area) were selected for this study (Figure1); these farms represented approximately 2·4% of all herds in the state. All sampled farms were registered with the agency responsible for the state health surveillance. The number of farms was calculated based on the premise that each repetition has a maximum weight of 3·33% in the sample,^17^ and the response was expressed as a percentage. At each farm, blood samples were randomly collected from 20 animals in each production cycle category: breeding animals (sows and gilts), farrowing crate (2–3 weeks), nursery (4–7 weeks), grower pigs (8–14 weeks), and finisher pigs (15–16 weeks). In total, there were 100 samples per farm and 3000 animals in the study. All farms were farrow-to-finishing operations, with no SIV vaccination history, and most farms utilized all-in-all-out production systems. The farms had between 160 and 1950 sows, and three farms had experienced respiratory disease outbreaks with swine influenza diagnostic confirmed between 3 and 6 months prior to the sample collection. This study was conducted under the approval of the Universidade Federal de Minas Gerais ethics committee. All herd owners provided consent for the use of the sera.

### Virus and control

The samples were tested for hemagglutination inhibition (HI) activity against the H1N1 pandemic strain (A/swine/Brazil/11/2009) and the H3N2 IAV (A/swine/Iowa/8548-2/98) reference strain. The same viruses were used in the back titration in each plate for the HI assay, and phosphate-buffered saline (PBS; pH 7·4) was used as a negative control.

### Hemagglutination inhibition

The HI test was performed as previously described,^18^ with some modifications. The sera were treated with 20% kaolin suspension and 0·5% rooster red blood cell (RBC) suspension to remove non-specific inhibitors and natural serum agglutinins. The initial serum dilution was 1:20 using PBS; then, each sample was diluted twofold to a final dilution of 1:640 in 96-well V-bottom plates. The HI antibody titer of each sample was determined as the reciprocal of the highest dilution in which no hemagglutination was observed. Samples with an HI titer ≥40 were considered to be positive. Titers were classified as follows: ≤20, negative; 40–80, low; 160–320, medium; and ≥640, high. Values were expressed by mean log_2_ antibody titers. Means log_2_ antibody titers are equivalent to HI titers: 4·32 = 20; 5·32 = 40; 6·32 = 80; 7·32 = 160; 8·32 = 320; and 9·32 = 640. A herd was considered positive when at least one of the animals was seropositive.

## Results

In this study, the percentages of animals with antibodies against H1N1pdm09 and H3N2 SIV were 26·23% and 1·57%, respectively, considering the 3000 samples tested. The percentages of seropositive herds for both viruses were 96·6% and 13·2%, respectively. The HI results per area and virus are summarized in Table1.

**Table 1:** Seroprevalence of H1N1pdm09 and H3N2 SIV for herds and animals for all studied areas in Minas Gerais state, Brazil

Areas	H1N1pdm09		H3N2 SIV	
	Herds Positive (%)	Animals Positive (%)	Herds Positive (%)	Animals Positive (%)
1	6 (20%)	95 (3·17%)	1 (3·3%)	18 (0·6%)
2	8 (26·6%)	348 (11·6%)	1 (3·3%)	2 (0·07%)
3	9 (30%)	190 (6·33%)	0	0
4	6 (20%)	154 (5·13%)	2 (6·6%)	27 (0·9%)
Total	29 (96·6%)	787 (26·23%)	4 (13·2%)	47 (1·57%)

Areas: 1 = Belo Horizonte Metropolitan area; 2 = Zona da Mata; 3 = South/Southwest; 4 = Triângulo Mineiro/Alto Paranaíba.

**Figure 2:**
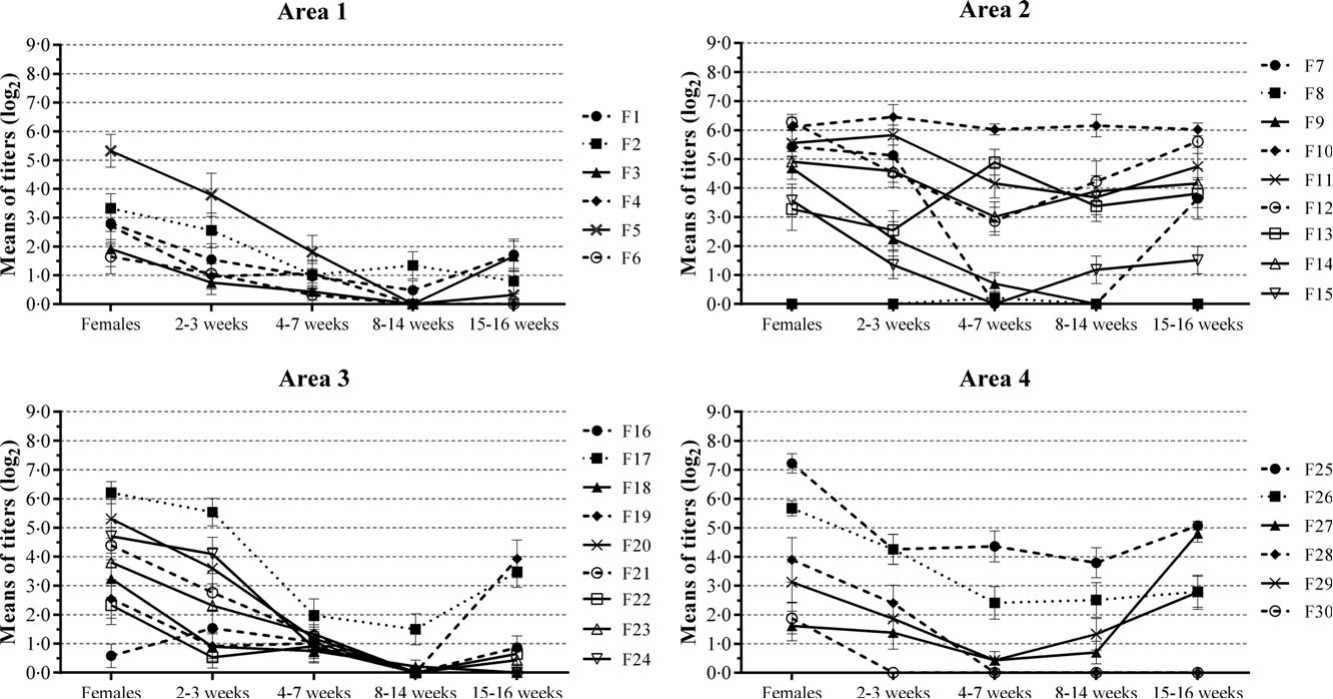
Serological profiles to H1N1pdm09 virus per area in all studied farms in Minas Gerais state, Brazil. Areas: 1 = Belo Horizonte Metropolitan area; 2 = Zona da Mata; 3 = South/Southwest and 4 = Triângulo Mineiro/Alto Paranaíba. Values are shown as log_2_ transformed mean titers with standard error of the means.

The number of pigs with anti-H1N1pdm09 antibodies varied between the farms. Serological profiles against this antigen in each studied area are shown in Figure2. One farm (F16) from area 3 had a higher mean antibody titer in the farrowing crate group than in breeding animals (female group) (Figure2). This situation also occurred at two farms from area 2. The serological profiles from areas 1, 3, and 4 followed a characterized pattern of antibody profiles in which the antibody titers were higher in the female group and decreased through 8–14 weeks of age when seroconversion by natural infection normally occurs. In contrast, the serological profiles from area 2 varied greatly, and in some farms, seroconversion occurred earlier (4–7 weeks) in the production system (Figure2). In addition, some farms had detectable antibody levels throughout all ages, and the means of the antibody titers did not reach negative values, suggesting that the virus had recently circulated in all groups at these farms. Furthermore, F10 showed relatively constant mean antibody titers throughout the production cycle, suggesting virus circulation in all categories. Additionally, one farm (F8) had no anti-H1N1pdm09 antibodies at all ages, suggesting that the H1N1pdm09 virus was not circulating in the herd at that moment.

**Figure 3:**
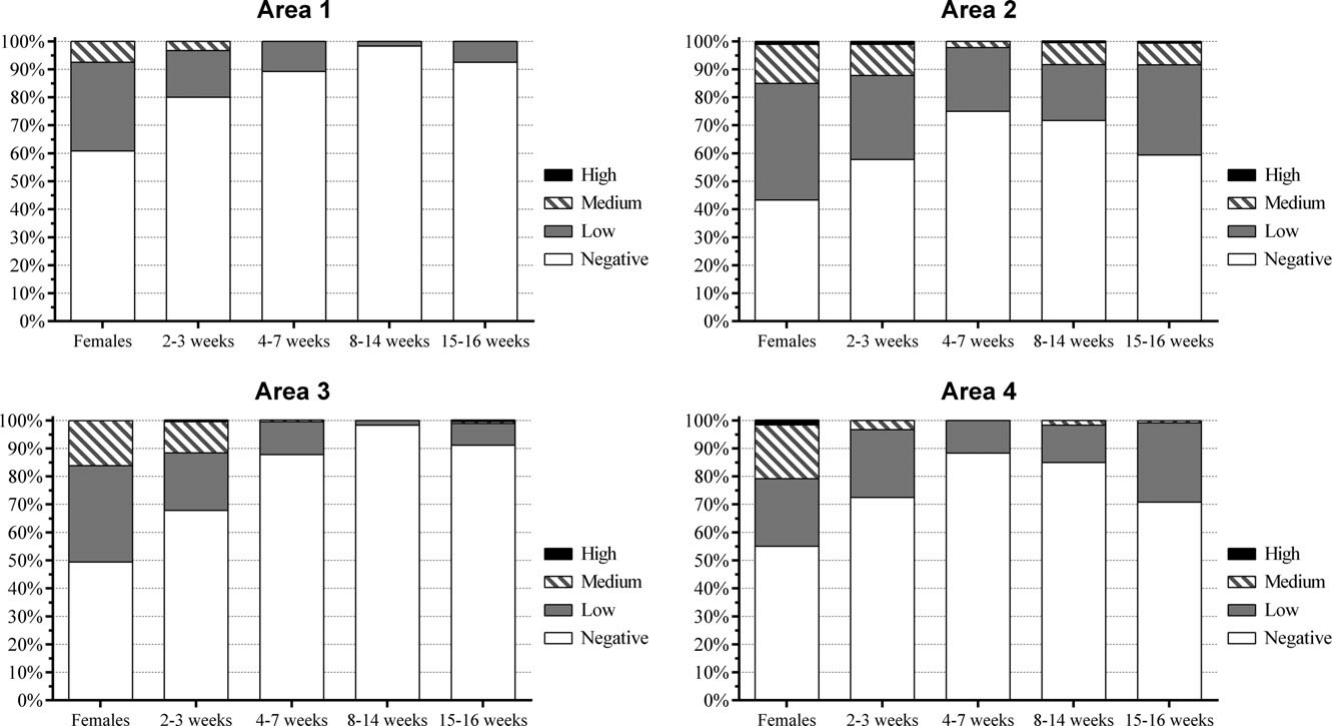
Distribution of H1N1pdm09 antibodies in all studied areas, according to the production cycle category. Areas: 1 = Belo Horizonte Metropolitan area; 2 = Zona da Mata; 3 = South/Southwest and 4 = Triângulo Mineiro/Alto Paranaíba.

The percentage of antibodies in each category of the production cycle also varied between the farms and studied areas (Figure3). At one farm (F30), only the females demonstrated anti-H1N1pdm09 antibodies, and all other categories were seronegative for this virus. Additionally, 28 of 30 farms (93·3%) had at least one piglet from the farrowing crates cycle with at least low antibody titers against the pandemic virus. Grower and finisher pigs from 17 farms were all seronegative for H1N1pdm09. A total of 98·33% of grower pigs in areas 1 and 3 were seronegative for H1N1pdm09 virus, and the percentage of seronegative animals in the finisher pig cycle was lower than that in the grower pig cycle for all studied areas. Overall, the percentage of pigs seronegative for H1N1pdm09 virus increased with age, and the highest values were observed in nursery pigs (areas 2 and 4) and grower pigs (areas 1 and 3) (Figure3). Interestingly, one area 1 farm (F6) and two area 3 farms (F21 and F22) had respiratory outbreaks, with influenza virus confirmed as a primary causative agent, a few months prior to sample collection. At that time, clinical disease occurred mostly in nursery and grower pigs, thus corroborating our findings demonstrating susceptibility at these ages.

**Figure 4:**
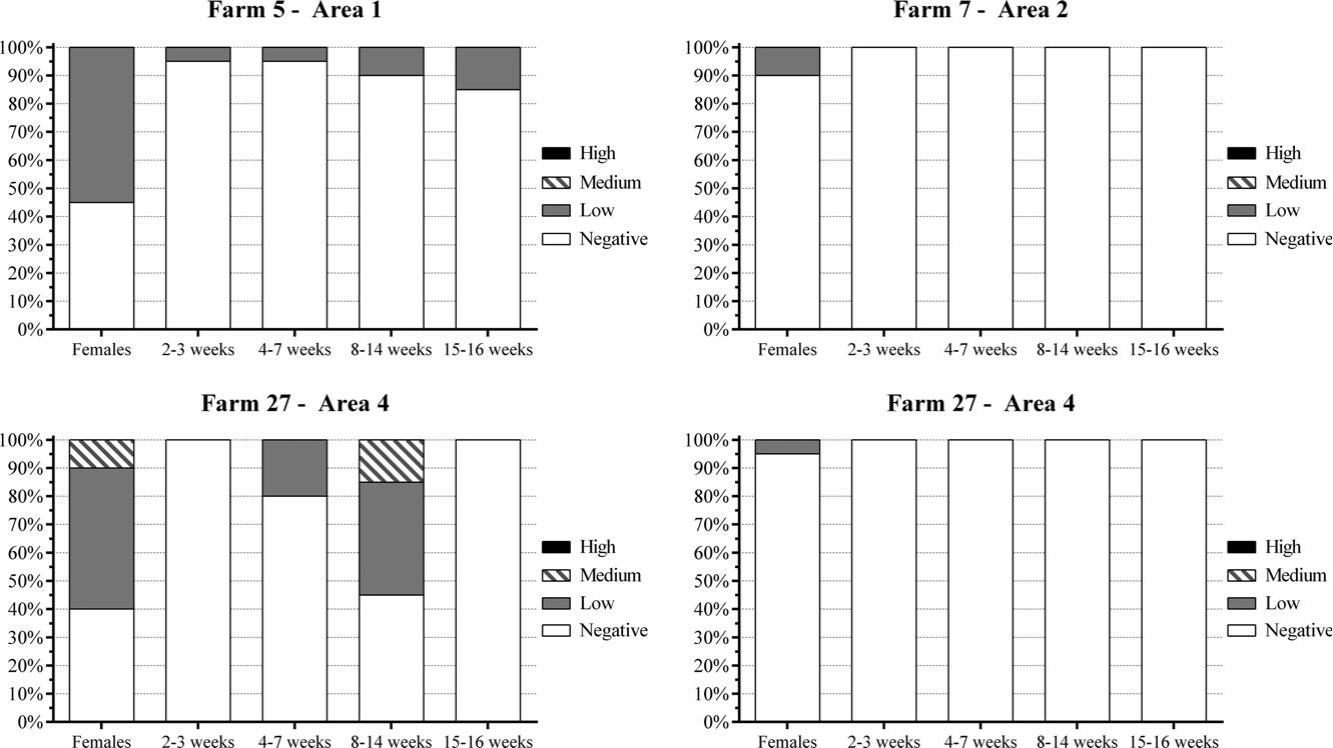
Distribution of anti-H3N2 antibodies in all seropositve farms, according to production cycle category. 1 = Belo Horizonte Metropolitan area; 2 = Zona da Mata; 3 = South/Southwest and 4 = Triângulo Mineiro/Alto Paranaíba.

Figure4 shows the antibody distribution at all four farms with animals that were seropositive for H3N2 SIV virus. The most varied profile of antibodies against H3N2 SIV virus was observed at Farm 27 (area 4), at which the pigs showed low to medium antibody levels. Farms 7 (area 2) and 29 (area 4) had only two and one females, respectively, with anti-H3N2 SIV antibodies. At these farms, no animals were seropositive for H3N2 SIV virus in the other categories. In Farm 5 (area 1), 18 of 100 pigs had low levels of antibodies against H3N2 SIV virus, and 82% had no antibodies against this virus.

## Discussion

Influenza is a disease that is associated with economic losses in swine herds. SIV is widespread in swine worldwide, and many countries have described virus circulation and antibody prevalence in pig populations. In Brazil, few studies have shown the presence of influenza virus in the swine population.^10^,^11^,^13^–^15^ However, the anti-influenza antibody prevalence after the H1N1 pandemic and the susceptibility of pigs in farrow-to-finish farms has not been extensively studied in the country. The detection of anti-influenza antibodies in most of the farms suggests virus circulation, once Brazil had no approved vaccination program against influenza while this experiment was being performed. Virus circulation is also suggested in this study because of the variation of antibody titers between the farms and across the sampled categories, which may be caused by seroconversion at some farms.

In this study, we found that 26·2% and 1·57% of sampled animals and 96·6% and 13·3% of herds were seropositive for H1N1pdm09 and H3N2 IAV, respectively. The rates of animals with anti-influenza antibodies found in this study were lower than those previously reported for many countries. One study reported that 66·3% of the animals at swine farms in the United States had antibodies against subtype H1; a lower rate (33·7%) was detected for antibodies against the H3 subtype.^19^ Another study investigating the seroprevalence of antibodies against H1N1, H3N2, and H1N2 in sows from European herds detected seroprevalence rates that were >50% for H1N1 and H3N2 in Belgium (80·8% and 53·8%, respectively) and in Germany (70·8% and 58·6%, respectively). However, the prevalence rates were lower in Italy (46·4% and 41·7%, respectively) and Spain (38·5% and 38·0%, respectively).^20^ The seroprevalence of H1 and H3 subtypes at farrow-to-finish and specialized finishing farms from Netherlands was investigated; at the farrow-to-finish farms, 44·3% of the animals were seropositive for H1, and 6·6% of the animals were seropositive for H3.^21^ Although the prevalence of anti-influenza antibodies against H1 viruses has been higher than that of H3 viruses in most countries, comparing these prevalences is challenging when analyzing vaccinated and non-vaccinated herds because the circulation of influenza viruses at these farms may assume different routes, depending on the type of production system.^21^ Furthermore, although the H3N2 used in our study has been isolated in pigs from North America in 1998, there is a relative cross-protection between viruses with the same type of hemagglutinin. Using a H3N2 from different country might be one factor to explain the low prevalence for antibodies against H3N2 in our study. Despite of that, due the cross-protection, our results could be useful to show H3N2 general circulation in Brazil.

Some Brazilian studies have also detected anti-influenza antibodies at pig farms. A seroprevalence study of a farm in São Paulo found a prevalence of 85·3% for both H1N1 and H3N2 viruses;^11^ however, rates of 2·2% and 16·3%, respectively, were found for the same viruses in a study of several Brazilian states.^10^ In another study, a prevalence of 20·1% was reported for the H3N2 virus in a herd in Paraná state.^13^ Although the prevalences in these Brazilian studies were related to human viruses, our rates were not similar. In 2009, a seroprevalence study investigated the prevalence of anti-swine influenza virus antibodies in sows from 17 farrow-to-finish farms in Minas Gerais state and found rates of 44·5% and 10·1% for swine H1N1 and H3N2 viruses, respectively.^12^ In this case, although the prevalence was higher than that found in our study, it aligns more closely than those found in other Brazilian states. In contrast, a higher prevalence of antibodies against H3N2 virus was found in a herd from southern Brazil,^13^ suggesting that there may be differences in the circulating serotypes of influenza virus in different regions of Brazil.

Vaccination against influenza virus was not regulated in Brazil for the duration of this study; therefore, the presence of anti-influenza antibodies in Brazilian pigs detected herein suggests natural infection. Despite viral circulation, we found that one and 26 farms were seronegative for H1N1pdm09 and H3N2 viruses, respectively. At these farms, as well as at farms with low antibody levels, the pigs were still susceptible to influenza virus infection and clinical disease because of their lack of immunity against the virus. The introduction of influenza virus into a herd is usually associated with moving and the introduction of new animals.^22^ Moreover, a higher seroprevalence of anti-influenza antibodies has been associated with a greater density of pigs, which may lead to increased contact and also facilitate airborne transmission between herds.^23^ Minas Gerais is the fourth largest pork producing state in Brazil, and 22·6% of its commercial farms have ≥100 sows,^24^ which suggests that a high pig density in the state may be associated with risk factors for influenza virus infection, particularly in negative herds, in which pigs are more vulnerable.

In this study, the female and farrowing crate groups had the highest percentages of seropositive animals. Females more frequently had the greatest mean antibody titers and the highest number of animals with low and medium titers, followed by the farrowing crate group. For females, these results may indicate frequent exposure to influenza virus and continuous viral circulation because they remain in the production system longer. The occurrence of high levels of antibodies against influenza virus in farrowing piglets, in association with the decrease of antibody levels over time, is similar to that in a study of serological profiles in farrow-to-finish farms in Taiwan.^25^ In that study, the lowest maternally derived antibodies against influenza virus were observed at 3–9 weeks of age, suggesting that animals are more susceptible later in the production cycle. Maternally derived antibodies are able to partially protect piglets against clinical disease, but not against infection,^26^ and suckling piglets may become infected and shed virus in their secretions even in the presence of passive antibodies.^3^ In such cases, piglets with maternally derived antibodies could play an important role in viral spread in the herd.

In this study, most grower and finisher pigs were seronegative for H1N1pdm09 and H3N2 viruses. The levels of anti-influenza antibodies decrease over time, and in some studies, maternally derived antibody levels were undetectable in animals between nine and 10 weeks of age.^25^,^27^ These results may explain why pigs later in the nursery age and early in the growing period are more vulnerable to influenza virus infection. Moreover, the timing of influenza infection may vary based on the type of production system.^21^ A study comparing the timing of infection in finisher pigs in non-vaccinated farrow-to-finish herds and specialized finishing herds showed that infection was highest at the beginning of the finishing period in farrow-to-finish herds, while in finishing herds, the incidence of influenza infections was highest in the end of the same period.^21^ Differences in the time of influenza infection occurrence may require different preventive measures, and because passive antibodies may interfere in the immune response to vaccination or infection, vaccination strategies must be analyzed to improve protection against influenza infection. Based on our results, it is not possible to suggest a single vaccination program against influenza virus because susceptible animals were generally detected in all categories of the production system. Thus, some strategies, such as female-only vaccination or female vaccination followed by vaccination of pigs at 7–8 weeks, could be helpful, depending on the immunity of the herd.

A few months prior to the sample collection, some farms had outbreaks of respiratory disease in nursery and grower pigs due to influenza virus infection. According to our results, the percentage of seronegative pigs for H1N1pdm09 virus increased with age, and the highest values were observed in nursery and grower pigs. Overall, influenza virus circulates in the herds and may cause disease in susceptible pigs^21^ regardless of age. The presence of seronegative animals in the nursery and grower phases (after reported outbreaks in these ages) suggests that influenza virus was circulating in the farms but that during the sampling, the virus was not circulating in the nursery and grower pig categories.

The percentages of seropositive pigs for H1N1pdm09 and H3N2 SIV found in this study differed between them, and H1N1pdm09 showed higher circulation in herds in Minas Gerais state. The serological profiles differed for both viruses and among geographical areas, suggesting a high variety of viral circulation across the state and resulting in seronegative animals that were susceptible to influenza infection and respiratory disease outbreaks. Little information is available regarding the influenza subtypes circulating in swine in the country, and because the lack of cross-protection between different subtypes is one of the obstacles preventing the development of efficacious vaccines and prevention of influenza infection in swine, more studies are needed to improve the knowledge of viral circulation and to determine the best strategies for preventing economic losses related to influenza virus infection in Brazil. Studies involving serological surveillance for the influenza virus in other regions in Brazil, detection of endemic viruses circulating in swine herds and monitoring the genetic evolution of these samples by sequencing could be useful to provide information about molecular epidemiology of SIV and provide data for vaccine production and control of influenza virus in Brazil.
